# Neuronal migration abnormalities and its possible implications for schizophrenia

**DOI:** 10.3389/fnins.2015.00074

**Published:** 2015-03-10

**Authors:** Kazue Muraki, Kenji Tanigaki

**Affiliations:** Shiga Medical Center, Research InstituteMoriyama, Shiga, Japan

**Keywords:** schizophrenia, GABAergic interneuron, mouse models, Neuregulin1, DISC1, 22q11 deletion syndrome, Dgcr8, Cxcr4

## Abstract

Schizophrenia is a complex mental disorder that displays behavioral deficits such as decreased sensory gating, reduced social interaction and working memory deficits. The neurodevelopmental model is one of the widely accepted hypotheses of the etiology of schizophrenia. Subtle developmental abnormalities of the brain which stated long before the onset of clinical symptoms are thought to lead to the emergence of illness. Schizophrenia has strong genetic components but its underlying molecular pathogenesis is still poorly understood. Genetic linkage and association studies have identified several genes involved in neuronal migrations as candidate susceptibility genes for schizophrenia, although their effect size is small. Recent progress in copy number variation studies also has identified much higher risk loci such as 22q11. Based on these genetic findings, we are now able to utilize genetically-defined animal models. Here we summarize the results of neurodevelopmental and behavioral analysis of genetically-defined animal models. Furthermore, animal model experiments have demonstrated that embryonic and perinatal neurodevelopmental insults in neurogenesis and neuronal migrations cause neuronal functional and behavioral deficits in affected adult animals, which are similar to those of schizophrenic patients. However, these findings do not establish causative relationship. Genetically-defined animal models are a critical approach to explore the relationship between neuronal migration abnormalities and behavioral abnormalities relevant to schizophrenia.

## Introduction

Schizophrenia is a chronic psychiatric disorder with a strong genetic component. Twin studies indicated that the heritability for schizophrenia is estimated to be 0.81 (Sullivan et al., 2012). Most of genetic linkage studies failed to identify highly-shared risk alleles due to the complexity of genetic architecture of schizophrenia except for DISC1 (Millar et al., 2000). Many combinations of different gene variants cause genetic risk of schizophrenia. Genome-wide association studies have identified many schizophrenia susceptibility candidate genes. Most of such common variants confer only slight increase in risk for schizophrenia (odds ratio < 1.2) (Ripke et al., 2013), and often failed to be replicated. Some of them, Neuregulin1, ErbB4, and Reelin are involved in the regulation of neuronal migration. On the other hand, rare and *de novo* chromosomal microdeletion or microduplication [copy number variations (CNVs)] have been implicated in schizophrenia (Levinson et al., 2011; Rees et al., 2014). Such CNVs are from thousands to millions nucleotides and contain many genes and their odds ratios are high (2~20) compared with common variants. 22q11.2 deletion syndrome (22q11DS) is the most frequent known genetic cause of schizophrenia (Pulver et al., 1994). However, it remains to be elucidated how combinations of these genetic variants play pathogenic roles of schizophrenia.

Schizophrenia is believed to result from embryonic developmental abnormalities not from neuronal degenerations (Weinberger, 1987; O'Connell et al., 1997). Cytoarchitectural abnormalities were reported in the entorhinal cortex (Jakob and Beckmann, 1986; Arnold et al., 1991, 1997; Falkai et al., 2000) and the subcortical white matters in schizophrenia (Akbarian et al., 1993a,b, 1996). Decreased neuronal density in the superficial white matter and increased density in the deep white matter suggest neuronal migration defects in schizophrenia. However, other studies failed to replicate these findings (Akil and Lewis, 1997; Krimer et al., 1997; Bernstein et al., 1998; Beasley et al., 2002, 2009), suggesting these abnormalities might be too subtle to be detected without special methods. Prior human brain imaging studies also have indicated reduced cerebral volume, ventricular enlargement, and reduced hippocampal volume in schizophrenia (Shenton et al., 2001).

The development of the human cerebral cortex is similar to that in the mouse (Rakic, 2009; Hansen et al., 2013; Ma et al., 2013), which enabled investigation of the functions of schizophrenia susceptibility candidate genes in neuronal developments. The mammalian cerebral cortex consists mainly of excitatory glutametergic and inhibitory GABAergic neurons. Glutamatergic neurons are generated from neural progenitors in the dorsal forebrain (Glover et al., 2009; Rakic, 2009), whereas most of inhibitory neurons are thought to derive from the ventral pallium: medial, lateral and caudate ganglionic eminence (MGE, LGE, and CGE) (Hansen et al., 2013; Ma et al., 2013). Two types of migration are observed in the cortex. One is radial migration of glutamatergic neurons from the underlying ventricular zone along the radial glial fiber, while the other is tangential migration from the ventral forebrain of GABAergic interneurons (Corbin et al., 2001; Marin and Rubenstein, 2003). However, the differences between the human and mouse cortices are also reported. In the mouse, about 70% of cortical interneurons generate from the MGE and ~30% are from the CGE (Miyoshi et al., 2010). In contrast, more than half of interneurons derive from the CGE in the human (Hansen et al., 2013). The human cortex showed a much higher diversity in the interneuron types compared with that of the rodents (Feldman and Peters, 1978). In spite of the limitations caused by these differences, animal models are still valuable to elucidate the roles of neuronal migration deficits in the pathogenesis of schizophrenia. Many genetically-modified animal models with construct validity and cell-specific gene modification technique are available. The great advantage of rodent model to study schizophrenia is that we can establish a causal relationship between genetic abnormalities, neuronal developments, and behavioral abnormalities. Here we review a group of studies using rodent models which give insights into the pathogenesis of schizophrenia.

## Developmental neuronal disruption model of schizophrenia

Perinatal insult of neuronal development can cause anatomical and behavioral deficits similar to human schizophrenic patients. One of the examples is a gestational day 17 (GD17) methylazoxymethanol acetate (MAM) administration rat model (Grace and Moore, 1998; Flagstad et al., 2004; Gourevitch et al., 2004; Paredes et al., 2006). MAM is a mitotic toxin and MAM administration specifically disrupts proliferating region. GD17 MAM treatment results in specific subtle reductions in the volume of prefrontal cortex (PFC) and hippocampus (HP), heterotopias in the HP resulting from neuronal migration deficits (Le Pen et al., 2006; Moore et al., 2006), which are characteristics of schizophrenia (Kovelman and Scheibel, 1984; Shenton et al., 2001; Heckers, 2004; Honea et al., 2005). GD17 MAM-treated rats also display decreased density of parvalbumin (PV)-positive interneurons in medial PFC and HP (Lodge et al., 2009). Interestingly, postmortem studies of human schizophrenia have shown decreased expression of PV and the 67 KDa isoform of glutamic acid decarboxylase (GAD67), which is an enzyme responsible for GABA synthesis, in PFC of schizophrenia subjects (Akbarian et al., 1995; Volk et al., 2000; Hashimoto et al., 2003; Fung et al., 2010). However, no difference is observed in the density of PV-positive interneurons in schizophrenia (Hashimoto et al., 2003). PV-positive interneurons are known to be indispensable for synchronized firing of excitatory pyramidal neurons in gamma frequencies (30–80 Hz), which plays essential roles for cognitive functions (Howard et al., 2003). Altered gamma oscillation activity and cognitive deficits have been reported in schizophrenia (Cho et al., 2006; Minzenberg et al., 2010). Consequently, PV-positive interneuron deficits are thought to be the cause of impairments of gamma oscillation and cognition in individuals with schizophrenia (Lewis et al., 2012). MAM-treated rats also show behavioral deficits in prepulse inhibition (PPI), which reflects an inability to filter out irrelevant sensory information, and working memory task (Flagstad et al., 2005; Le Pen et al., 2006; Moore et al., 2006), which are typical symptoms of schizophrenia in humans (Braff and Geyer, 1990; Liddle and Morris, 1991; Goldman-Rakic, 1994; Swerdlow et al., 1994; Nuechterlein et al., 2004). Furthermore, electrophysiological studies have shown that enhanced activity of ventral HP leads to dopaminergic neuronal activation in MAM-treated rats (Lodge and Grace, 2007). Again, these altered hippocampal activities are also observed in human schizophrenic patients (Medoff et al., 2001; Schobel et al., 2009). These abnormalities can be normalized by administration of α5GABA A receptor positive allosteric modulator, SH-053-2′F-R-CH3 (Gill et al., 2011), suggesting the involvement of GABA in embryonic MAM treatment-induced deficits. The MAM model provides a direct evidence that subtle embryonic disruptions of neuronal development result in behavioral alterations disorders, although the etiology is absolutely different from that of schizophrenia in humans.

## Neuregulin-ERBB signaling

Neuregulins are a large family of epidermal growth factor (EGF)-like proteins and play divergent roles both in neuronal development and in the neuronal activity homeostasis in the mature central nervous system. Several genetic linkage studies have shown Neuregulin1 (NRG1) as a strong candidate gene for schizophrenia (Badner and Gershon, 2002; Stefansson et al., 2002; Lewis et al., 2003). Some GWASs also support the hypothesis (Li et al., 2006; Munafo et al., 2006; Shi et al., 2009; Agim et al., 2013), although it has not been confirmed by a recent mega-analysis in which international consortia combined the resources to maximize the sample size and identified more than 100 candidate genes for schizophrenia with high levels of statistical significance (Schizophrenia Working Group of the Psychiztric Genomics Consortium, 2014). Most of schizophrenia-associated single nucleotide polymorphisms (SNPs) in NRG1 are localized in the 5′ and 3′ region of the gene. Some of them are associated with the expression level of NRG1 (Law et al., 2006; Weickert et al., 2012). A receptor of NRG1, ERBB4 is also associated with schizophrenia (Benzel et al., 2007; Law et al., 2007; Shi et al., 2009; Agim et al., 2013). It has been reported that a rare chromosome micro-deletion in a schizophrenic patient disrupts this gene, resulting in a truncated protein similar to dominant-negative ERBB4 (Walsh et al., 2008).

Nrg1 generates six types and at least 30 isoforms owing to multiple promoters and alternative splicing (Mei and Nave, 2014). Most pro-Nrg1 isoforms are transmembrane proteins and N-terminal domains containing EGF-like domain are released out of the cell after undergoing proteolytic processing except for TypeIII Nrg1 (cysteine-rich-domain containing Nrg1 (CRD-Nrg1)). This released mature Nrg1 activates ErbB receptor tyrosine kinase such as ErbB2/ErbB3 heterodimer and ErbB4 homodimers. Nrg1 regulates migration of excitatory glutamatergic neurons and γ-aminobutyric acid (GABA)-producing interneurons in the embryonic cortex. Nrg1 promotes the maintenance of radial glial cells in the cortex and induces elongation of radial fiber, which are essential for the radial migration of cortical excitatory neurons and cerebellar granule cells (Anton et al., 1997; Rio et al., 1997). NRG1 is also critical for interneuronal tangential migration (Flames et al., 2004; Li et al., 2012). ErbB4 is expressed in interneuronal progenitors migrating from the MGE to the cortex (Yau et al., 2003; Flames et al., 2004). Type III Nrg1 is expressed in lateral ganglionic eminence, and Type I and II Nrg1 (immunoglobulin (Ig)-domain containing Nrg1 (Ig-Nrg1)) are expressed in the embryonic cortex (Flames et al., 2004). Diffusible Type I and II Nrg1 in the cortex are thought to attract ErbB4-expressing interneurons along a permissive corridor of Type III Nrg1 (Flames et al., 2004), although this model is challenged. In another model, Nrg1, and Nrg3 have been proposed to be repellants for migrating interneurons (Li et al., 2012). Loss of ErbB4 cuases embryonic lethality due to failed development of myocardial trabeculae, which made it difficult to characterize the functions of ErbB4 signaling in interneuronal migration (Gassmann et al., 1995; Kramer et al., 1996). However, heart-rescued ErbB4 knockout mice with cardiac-specific ErbB4 transgene displayed decreased number of GABAergic interneurons in the postnatal cortex (Flames et al., 2004; Fisahn et al., 2009; Li et al., 2012), which clearly showed the essential roles of Nrg1/ErbB4 signaling in interneuronal migration.

Cell-specific gene modification techniques are now starting to elucidate a link between Nrg1/ErbB4 signaling and pathophysiology of schizophrenia. The gain and loss of function of Nrg1/ErbB4 signaling were examined because postmortem studies of schizophrenia reported both increased and decreased NRG1/ERBB4 signaling in schizophrenic patients (Silberberg et al., 2006; Law et al., 2007; Weickert et al., 2012; Joshi et al., 2014). Transgenic mice overexpressing Type I Nrg1 showed deficits in PPI and contextual fear conditioning, and hyperlocomotion (Deakin et al., 2009, 2012; Yin et al., 2013; Luo et al., 2014). If the overexpression of Nrg1 was switched off in adult mice, its effects were reversible (Yin et al., 2013; Luo et al., 2014). The influence of Nrg1 overexpression on neuronal development remains to be elucidated. Nrg1 or ErbB4 heterozygous mice and conditional knockout mice also displayed various behavioral abnormalities: locomotor hyperactivity in open field (OF), impairment in Prepulse inhibition (PPI), and fear conditioning (Stefansson et al., 2002; Golub et al., 2004; Boucher et al., 2007; O'Tuathaigh et al., 2007, 2010; Chen et al., 2008, 2010; Duffy et al., 2008; Ehrlichman et al., 2009; Shamir et al., 2012; Del Pino et al., 2013; Pei et al., 2014)(Table 1). Nrg1 heterozygous mice and heart-rescued ErbB4 knockout (KO) mice showed decreased number of cortical PV interneurons (Fisahn et al., 2009; Neddens and Buonanno, 2010; Shamir et al., 2012; Pei et al., 2014) (Table 1). However, PV interneuron-specific deletion of ErbB4 did not affect the number of cortical interneurons, which might be due to the slow turnover of ErbB4 (Fazzari et al., 2010; Shamir et al., 2012) (Table 1). A comparative behavioral analysis of ErbB4 KO and PV interneuron-specific ErbB4 KO mice demonstrated that PV interneuron-specific deletion is sufficient for hyperactivity and deficits in PPI. The only difference is that ErbB4 KO mice but not PV interneuron-specific ErbB4 KO mice exhibit reduced anxiety-like behaviors and deficits in cued and contextual fear conditioning (Shamir et al., 2012), which might be caused by developmental disorders in ErbB4-deficient interneurons.

**Table 1:** **Summary of mutant Nrg1/ErbB4 mouse models**.

**Gene**	**Locomotion**	**PPI**	**Learning and memory**	**Gross anatomy**	**Interneuron**	**References**
Nrg1^+/-^ (TM)	↑	→	ND	ND	ND	Boucher et al., 2007
	↑	↓	ND	ND	ND	Stefansson et al., 2002
	↑	ND	Y maze	Small ventricle	ND	O'Tuathaigh et al., 2007, 2010
Nrg1^+/-^ (TM)	→	→	contextual FC ↓ cued FC ↓	ND	PV/Gad67 in HC ↓ (western blotting)	Pei et al., 2014
Nrg1^+/-^ (TypeIII)	→	↓	T-Maze↓	Enlarged lateral ventricle	ND	Chen et al., 2008
Nrg1^+/-^	↑	→	ND	ND	ND	Duffy et al., 2008
(EGF)	→	→	Contextual FC ↓	ND	ND	Ehrlichman et al., 2009
ErbB4^+/-^	↑	→	ND	ND	ND	Stefansson et al., 2002
ErbB4^f/-^ (Nestin-Cre)	↓	ND	Morri Water Maze ↑ (hetero)	ND	ND	Golub et al., 2004
ErbB4^-/-^ (heart-rescued)	↑	↓	Contextual FC ↓ Cued FC ↓	ND	Number of PV neurons ↓	Shamir et al., 2012
ErbB4^f/f^ (PV-Cre)	↑	↓	Contextual FC → Cued FC →	ND	Number of PV neurons →	Shamir et al., 2012
ErbB4^f/f^ (PV-Cre)	ND	ND	Contextual FC ↓	ND	ND	Chen et al., 2010
ErbB4^f/f^ (Lhx6-Cre)	↑	↓	Y-maze ↓	ND	Number of PV neurons →	Del Pino et al., 2013

*OF, open field, PPI:prepulse inhibition; FC, fear conditioning; TM, transmembrane region; PV, parvalbumin; HC, hippocampus; ND, not determined*.

## Disrupted-in schizophrenia 1

The disrupted-in schizophrenia 1 (DISC1) gene was discovered at the breakpoint of inherited balanced chromosomal translocation in a Scottish family suffering from major depression, schizophrenia, and bipolar disorder (St Clair et al., 1990; Millar et al., 2000). Following linkage analysis and association studies demonstrated that DISC1 is significantly associated with schizophrenia, bipolar disorder and major depression (Ekelund et al., 2001, 2004; Macgregor et al., 2004; Hamshere et al., 2005; Hashimoto et al., 2006; Liu et al., 2006; Thomson et al., 2014), although it has not been confirmed by a recent mega-analysis of GWASs (Schizophrenia Working Group of the Psychiztric Genomics Consortium, 2014).

DISC1 is a scaffolding protein interacting with multiple proteins: nuclear distribution gene E homolog-like 1 (NDEL1), lissencephaly-1 (LIS1), phosphodiesterase 4B (PDE4B), glycogen synthase kinase 3β (GSK3β) and DIX domain-containing 1 (DIXDC1) (Ozeki et al., 2003; Millar et al., 2005; Taya et al., 2007; Mao et al., 2009). DISC1/DIXDC1 functions as a switch between neuronal proliferation and migration. DISC1/DIXDC1 binds to GSK3β and inhibits its activity leading to proliferation of neural progenitors through the inhibition of β-catenin degradation (Mao et al., 2009). CDK5 phosphorylation of DISC1 at S710 and DIXDC1 facilitates neuronal migration by dissociating DISC1 from GSK3β and promoting its binding with NDEL1 (Singh et al., 2010; Ishizuka et al., 2011). DISC1 variants associating with human brain structures and psychiatric phenotypes have been reported to impair this switching mechanism (Singh et al., 2011). Furthermore, knockdown of DISC1 also impairs interneuronal tangential migrations (Steinecke et al., 2012, 2014).

Two hypotheses are proposed on the pathophysiology of the disruption of the DISC1 gene: that the Scottish mutation decreases DISC1 expression and leads to haploinsufficiency; or that the Scottish mutation results in production of carboxy-terminal-truncated DISC1 (amino acids 1-598). This C-terminal truncated DISC1 functions as a dominant negative protein, and impairs microtubule dynamics by blocking interaction between DISC1 and dynein complex (Kamiya et al., 2005). Dynein complex contains LIS1 and NDEL1, and regulates coupling of the nucleus and centrosome, which is indispensable for radial migration of cortical excitatory neurons (Sasaki et al., 2000). Knockdown of DISC1 inhibits cortical neuronal cell migration (Kamiya et al., 2005; Kubo et al., 2010).

Acute knockdown of DISC1 using RNAi leads to drastic neuronal migration deficits. However, DISC1 KO mice (*Disc1^Δ2−3^/Δ2−3*) astonishingly showed almost normal cytoarchitectures of the cerebral cortex and the HP (Kuroda et al., 2011), whereas the number of PV-positive interneurons reduced in female *Disc1^Δ2−3^/Δ2−3* mice (Nakai et al., 2014). The phenotypes in the proliferation of neuronal progenitors have not been examined in DISC1 KO mice, which will provide important insight into the pathogenesis of DISC1 deficiency. Furthermore, *Disc1^Δ2−3^/Δ2−3* mice did not show schizophrenia-like phenotype but exhibited lower anxiety and higher impulsivity (Kuroda et al., 2011) (Table 2). Only female *Disc1^Δ2−3^/Δ2−3* mice exhibited enhanced responsiveness to methamphetamine and deficits in PPI (Kuroda et al., 2011). These milder phenotypes of *Disc1^Δ2−3^/Δ2−3* mice might be explained by a compensation mechanism after chronic loss of DISC1. N-nitroso-N-ethylurea (ENU) mutagenesis was utilized to generate missense mutations of *Disc1*. L100P mutant (*Disc1*^*L100P/L100P*^) mice showed reduced brain volume, reduced number of cortical neurons, altered distribution of cortical neurons, interneuonal migration deficits, and schizophrenia-like behavioral abnormalities, although the behavioral phenotypes were not confirmed by another group due to the difference in the genetic background (Clapcote et al., 2007; Lee et al., 2011, 2013; Shoji et al., 2012). 129S6/SvEv 25-bp deletion variant results in the production of a truncated isoform of DISC1 (amino acids 1-542) (Koike et al., 2006). C57BL/6J mice carrying the Disc1 gene from the 129S6/SvEv strain (Disc1^Δ25 bp/Δ25 bp^mice) exhibited enlarged ventricle and working memory deficits (Koike et al., 2006; Juan et al., 2014). Neuron-specific overexpression of the truncated DISC1 also resulted in drastic phenotypes: enlarged lateral ventricle, reduced number of PV-positive interneurons and schizophrenia-like behavioral abnormalities (Hikida et al., 2007; Pletnikov et al., 2008; Shen et al., 2008; Ayhan et al., 2011) (Table 2). Furthermore, a technique of inducible transgene expression enabled a specific expression of truncated DISC1 during only prenatal period, only postnatal period or both periods (Ayhan et al., 2011). Prenatal expression only led to decreased brain volume and decreased number of PV-positive interneurons. Enlarged lateral ventricle seems to be affected by postnatal expression of truncated DISC1. In contrast, enhanced responsiveness to psychostimulant required prenatal and postnatal continuous expression (Ayhan et al., 2011), which suggests that both neurodevelopmental abnormality and neuronal functional impairment caused by truncated DISC1 might be essential for pathogenesis of schizophrenia.

**Table 2:** **Summary of mutant Disc1 mouse models**.

**Gene**	**Locomotion memory**	**PPI**	**Learning and**	**Gross anatomy**	**Interneuron**	**References**
CaMK-	↑	↓	Y-maze →	Enlarged lateral ventricle	Number of PV neurons	Hikida et al., 2007
DN-DISC1 tg					↓	
BAC	→	ND	ND	Enlarged lateral ventricle	Number of PV neurons	Shen et al., 2008
DN-DISC1 tg					↓	
Inducible-CaMK	↑	→	ND	Enlarged lateral ventricle	Number of PV neurons	Pletnikov et al., 2008; Ayhan et al., 2011
DN-DISC1 tg					↓	
Disc1 ^Δ2-3^/Δ2-3	→	↓	Y-maze →	Normal	Number of PV neurons	Kuroda et al., 2011; Nakai et al., 2014
		(Female)			↓ (female)	
Disc1^L100P/L100P^	↑	↓	T-maze ↓	Brain volume↓	Deficits in the distribution of PV neurons	Clapcote et al., 2007; Lee et al., 2011, 2013; Shoji et al., 2012
Disc1 ^Δ25 bp/Δ25 bp^ (C57Bl6)	→	→	T-maze ↓	Enlarged lateral ventricle	ND	Koike et al., 2006; Juan et al., 2014

## 22q11 deletion syndrome

22q11.2 deletion syndrome (22q11DS) is the most frequent known genetic cause of schizophrenia (Pulver et al., 1994). 22q11DS accounts for about 1% of schizophrenia cases (Karayiorgou et al., 1995; Manolio et al., 2009). Prior brain imaging studies of human 22q11 DS have indicated reduced cerebral volume, ventricular enlargement and reduced hippocampal volume (Eliez et al., 2000, 2001; Chow et al., 2002; Simon et al., 2005). All of these brain anomalies have also been reported in schizophrenia (Shenton et al., 2001). All of the genes except for one gene in human 22q11.2 locus exist on mouse chromosome 16 (Puech et al., 1997). This has facilitated the generation of mouse models of 22q11 DS, which carry a hemizygous deletion of 22q11-related region of mouse chromosome16 (Lindsay et al., 1999; Paylor and Lindsay, 2006; Stark et al., 2008). These animal models show schizophrenia-related behavioral abnormalities such as working memory deficits, sensory information-processing deficits, and enhanced responsiveness to psychostimulants (Paylor et al., 2001; Stark et al., 2008; Earls et al., 2011; Kimoto et al., 2012), which are recognized as major deficits of schizophrenia (Elvevag and Goldberg, 2000; Green et al., 2000; Swerdlow et al., 2001). Animal models of 22q11DS showed reduced density of layer II–IV projection neurons in a medial PFC (Meechan et al., 2009), reduced volume of a perinatal HP dentate gyrus (Toritsuka et al., 2013), delayed migration of hippocampal dentate neuronal progenitors and cortical interneurons, and altered distribution of PV-positive interneurons (Meechan et al., 2009, 2012; Toritsuka et al., 2013), although it remains to be elucidated these deficits in neurogenesis lead to excitatory/inhibitory imbalance or not. Perinatal hippocampal DG and interneuronal migration abnormalities are caused by Cxcl12/Cxcr4 signaling deficits (Toritsuka et al., 2013). Cxcl12/Cxcr4 signaling might play pivotal roles in the pathogenesis of schizophrenia. Previous studies also suggest a possible involvement of Cxcl12/Cxcr4 signaling in the neurodevelopmental disorders of GD17 MAM-treated animal model of schizophrenia (Paredes et al., 2006). The expression of CXCL12 is decreased in olfactory neurons from sporadic cases with schizophrenia compared with normal controls (Toritsuka et al., 2013).

Among of genes deleted in 22q11DS, *Dgcr8* is a promising candidate gene for schizophrenia-related phenotypes. *Dgcr8* forms the microprocessor complex of microRNA (miRNA) with Drosha, which is essential for miRNA production. Overexpression of *Dgcr8* rescued interneuronal migration deficits of 22q11DS model mice, and the migration of hippocampal DG and interneuronal progenitors were also affected in*Dgcr8^+/−^* mice (Toritsuka et al., 2013). These observations demonstrated the important roles of *Dgcr8* in the pathogenesis of 22q11DS. miRNA–mediated regulation network fine tunes the balance of signaling and confers robustness to the system (Herranz and Cohen, 2010). miRNA-mediated regulation can buffer increases or reductions in gene dosage (Staton et al., 2011). Haplodeletion of *Dgcr8* causes 20–70% reduction of a specific subsets of mature miRNAs both in *Dgcr8* heterozygous and 22q11DS model mice (Stark et al., 2008). *Dgcr8* heterozygousity might uncover the effects of 22q11 microdeletion through the disruption of miRNA-mediated buffering effects. In mice, heterozygous deletion of *Dgcr8* alone showed working memory deficits, sensory information-processing deficits and some of neurodevelopmental abnormalities such as reduced cortical neuronal densities (Stark et al., 2008; Fenelon et al., 2011). The behavioral abnormalities and neurodevelopmental disorders of *Dgcr8^+/−^* mice are similar but some of them are milder than those of 22q11DS model mice,(Stark et al., 2008; Meechan et al., 2009; Fenelon et al., 2011), which might suggest that additional haplodeletion of other genes in 22q11-related regions might be required for the complete reconstitution of phenotypes of 22q11DS model mice. It remains to be elucidated whether behavioral abnormalities of 22q11DS model mice are directly caused by neuronal migration deficit and Cxcr4 signaling defects.

## Concluding remarks

Elucidating the relationship between neurodevelopmental abnormalities and the pathogenesis of schizophrenia would be exceptionally difficult. In order to dissect the complex causal relations, more sophisticated genetic manipulation would be required. Combination of various techniques such as conditional knockout, inducible transgene expression and virus-mediated gene delivery will enable cell type-specific and developmental stage-specific knockout or rescue experiments. In the future comprehensive profile of neurodevelopmental deficits-behavioral abnormalities will provide significant insights into mental disease pathogenesis of all these neurodevelopmental genes.

### Conflict of interest statement

The authors declare that the research was conducted in the absence of any commercial or financial relationships that could be construed as a potential conflict of interest.
